# Hyperhydricity Syndrome in In Vitro Plants: Mechanisms, Physiology, and Control

**DOI:** 10.3390/plants14243721

**Published:** 2025-12-05

**Authors:** Rajesh Barua, Abir U. Igamberdiev, Samir C. Debnath

**Affiliations:** 1 St. John’s Research and Development Centre, Agriculture and Agri-Food Canada, St. John’s, NL A1E 0B2, Canada; rbarua@mun.ca; 2 Department of Biology, Memorial University of Newfoundland, St. John’s, NL A1C 5S7, Canada; igamberdiev@mun.ca

**Keywords:** Hyperhydricity, apoplast, hypoxia, HPLC, ROS, MLM

## Abstract

Understanding the physiological characteristics of hyperhydric plantlets is ultimately necessary since hyperhydricity results in financial loss for in vitro plants from a commercial perspective. Although many studies report the possible causes and symptoms of hyperhydricity, knowledge of it remains limited. This review aims to provide an integrated overview of this phenomenon and outline the perspectives for its prevention. First, we summarize the factors of in vitro hyperhydricity, including gelling agents, growth regulators, vessel ventilation and gas exchange, light, and osmotic conditions. Second, we describe physiological and internal changes commonly observed in hyperhydric plants, such as ROS/ethylene imbalance, altered antioxidant capacity, defects in the cell wall, and lignification. Third, we outline ultrastructural characteristics and accumulate HPLC findings to recognize the metabolite profiles of hyperhydric plantlets. Fourth, we introduce emerging AI-assisted MLM (machine learning model) approaches to detect and optimize the culture parameters to prevent hyperhydricity. Finally, we evaluate the strategies for the protection of the culture from hyperhydric conditions. This structured overview intends to reduce hyperhydricity in commercial and research settings.

## 1. Introduction

**Figure 1:**
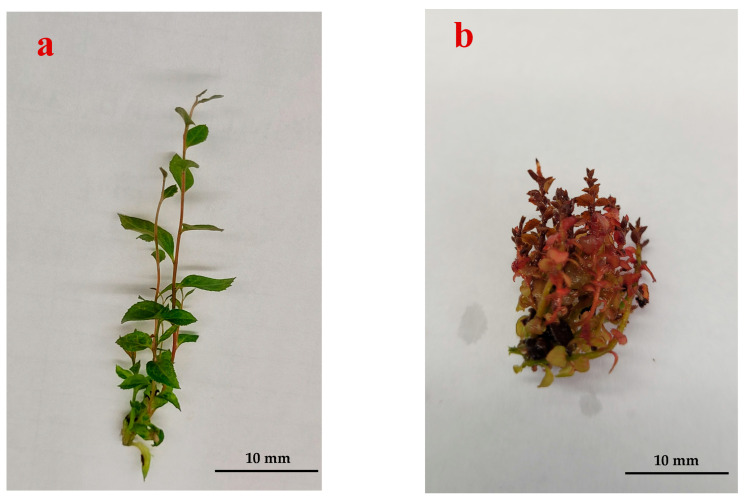
In vitro-cultured shoots in blueberry: (**a**) normal shoots; (**b**) hyperhydric shoots.

Hyperhydricity (previously known as vitrification), a condition characterized by physiological and morphological deformities [1,2,3,4] in tissue culture plants, was first reported 50 years ago. General morphological characteristics of hyperhydricity involve glassy, waterlogged tissue appearance, distorted growth, and so forth [5,6]. They mentioned that the explants were transparent because of high water content and aberrant chlorophyll levels. It was identified as a common phenomenon in over 200 plant varieties in tissue culture breeding [7,8,9]. The regeneration of hyperhydric plantlets (Figure 1) has resulted in financial losses [10,11]. It affects a lot of plantlets, including succulent, woody [12,13], and herbaceous plants, resulting in a decreased growth rate and poor survival ability [14,15]. Hyperhydricity is associated with reduced stomatal closure, altered leaf ultrastructure, wrinkled, curly, and brittle leaves, and oxidative stress [1,5,8,16]. According to Kevers et al. [17], cellular and subcellular level damage occurred in hyperhydric plantlets because of the increased polyamines (PAs) and lowered chlorophyll and protein levels. This is characterized by the overproduction of reactive oxygen species (ROS) causing oxidative stress [18,19,20]. The imbalance of the antioxidant mechanism contributes to essential metabolic changes in hyperhydric plantlets [21]. Furthermore, ROS changed the different expression levels of peroxidase, superoxide dismutase, glutathione S-transferase, glutathione peroxidase, and catalase, which showed significant variation in hyperhydric plantlets [1,8]. Several factors induce hyperhydricity, such as the addition of high concentrations of cytokinins [22], gelrite concentration enhancement, and changes in agar and sucrose concentrations [16,23,24]. Increased humidity, temperature, and light intensity can also induce hyperhydricity [16,23]. Hyperhydricity can cause substantial damage to plantlets and hinder their acclimatization during transfer to ex vitro circumstances [15].

Numerous strategies have been suggested to lessen hyperhydricity, including the use of filter paper or polyester screen rafts, altering agar concentration, pre-treatment with sucrose, and bottom cooling systems [4,25,26]. Other techniques involve using specific agents, silver ions, or hormone treatments [7,24,27,28,29] to reduce hyperhydricity. Furthermore, media solidified with gelrite and supplemented with arginine, ornithine, or spermidine [30] and silver ions supplied as silver thiosulfate or silver nitrate [31], and trichloroacetate [32], mitigated to hyperhydricity. The modified ratio of NH_4_^+^/NO_3_^−^ is the alternative method applied to reduce hyperhydricity. Some species exhibited high level of hyperhydricity, however, the application of deuterium-depleted water (DDW), was proved to reduce hyperhydricity such as Beta genus [33]. Reduced levels of water evaporation and diffusion molecules that contain heavier isotopes in the lead have an exaggerated ^18^O or ^2^H content in a stagnant state, preventing this glassy condition. However, limited research has focused on reducing hyperhydricity in plantlets. This article addresses current advances in the study of tissue culture plant stress (hyperhydricity), which led to the decline of the horticulture and agriculture sectors. This review also illustrates the internal and physiological changes resulting from the many factors influencing plant hyperhydric conditions.

## 2. Factors Inducing Hyperhydricity

**Figure 2:**
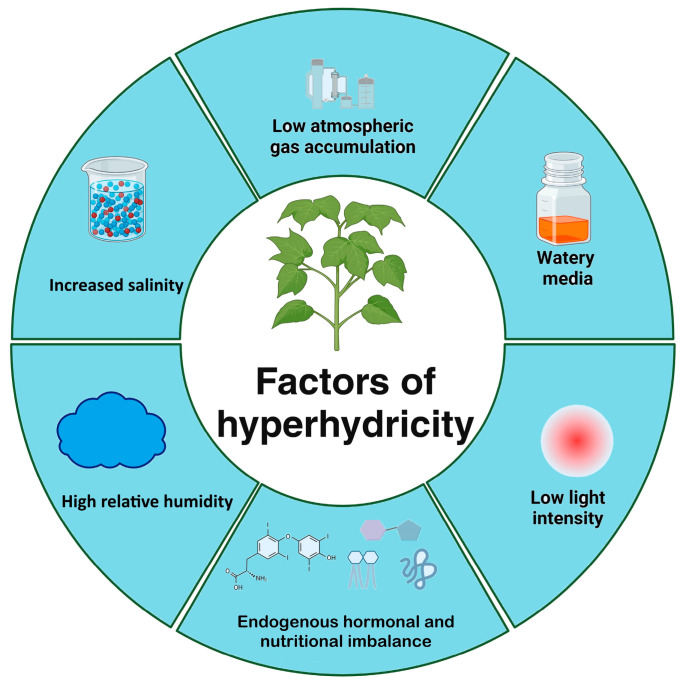
Factors of hyperhydricity.

Hyperhydricity occurs due to an inadequate local hormonal balance, which leads to an imbalance in the cell growth ratio between different cell types and rapid mesophyll cell vacuolation with excess water uptake [11,17]. This imbalance disrupts normal cell growth ratios, leading to rapid mesophyll cell vacuolation and excessive water uptake. As a result, H_2_O_2_ affects metabolism [34], while this process is indirectly influenced by ethylene. H_2_O_2_ accumulation disrupts redox homeostasis by interfering with NADPH-dependent pathways, leading to the oxidative inhibition of enzymes such as glyceraldehyde-3-phosphate dehydrogenase (GAPDH) and ultimately impairing glycolysis and carbon assimilation efficiency in regenerating tissues [35]. Ethylene generated under in vitro stress conditions enhances physiological disturbances that promote hyperhydricity in regenerating tissues. As demonstrated by Gao et al. [36], the *Dianthus chinensis* plantlet exhibited a high accumulation of ethylene and ROS. Abnormal nutrient balance and culture conditions induced hyperhydrocity in in vitro vegetative propagation of herbaceous and woody plants [6,37]. A comparatively high quantity of humidity was discovered to detect some potential factors that caused hyperhydricity (Figure 2). High minerals, carbohydrates, light intensity, and high growth regulators are critical factors for shoot malformation [5]. A high NH^4+^ to NO_3_ ratio in the nitrogen source disrupts the cellular osmotic balance, contributing to the hypertrophic characteristics of hyperhydric tissues. These conditions are prominent in plant tissue culture, particularly Caryophyllaceae [38]. Many reports revealed that hyperhydricity occurs due to the hypertrophic cells of plantlets, defective cell walls, and results of local hormonal imbalance [39].

## 3. Changes Under Hyperhydric Conditions

The distinctive features of hyperhydric plantlets (Figure 1) are observed by the deformation of vacuolated mesophyll cells, a thin cell wall, severe vascular tissue collapse, reduced stomatal aperture, less epidermal trichomes and glandular trichomes, stomatal malformation, membrane structure breakdown, and damaged chloroplasts (Table 1). These changes are clearly observed in flooded gum (*Eucalyptus grandis*), pink trumpet tree (*Handroanthus impetiginosus*) and teak (*Tectona grandis*) [40], and many micropropagated plants [6]. The overexpression of auxin-biosynthesis genes in mesophyll tissue leads to excess Indole-3-acetic acid (IAA), which inhibits root formation and triggers abnormal mesophyll expansion and water uptake. This hormonal imbalance, stimulated by high cytokinin levels, enhances vacuolation and the manifestation of hyperhydricity in newly formed shoots [41].

### 3.1. Structural Changes

**Figure 3:**
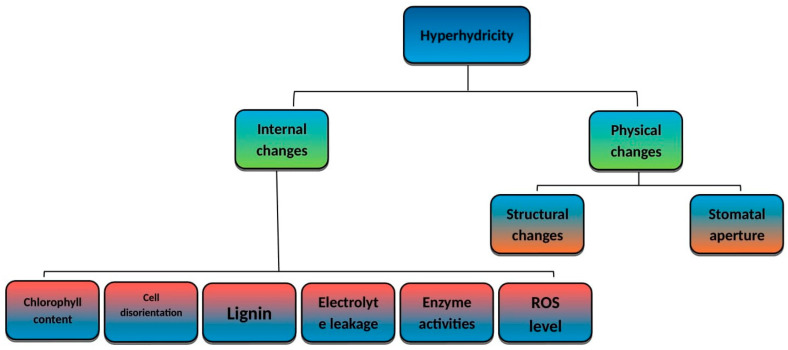
Morpho-physiological and biochemical changes under hyperhydric conditions.

Hyperhydric conditions cause significant morphological changes in plants (Figure 3). The plant cuticle plays an essential role in protecting the inner tissue; however, many research studies have revealed that impoverished epicuticular wax development is observed in hyperhydric plantlets [40]. Hyperhydricity is characterized by translucent, swollen, and brittle tissues, often recognized by thickened, water-soaked leaves and abnormal shoot morphology. Due to hyperhydricity, stomatal changes resulted in aberrant motions and impaired stomatal closure [3]. Leaves with high moisture showed deformed chloroplasts, less chlorophyll, and damaged granule shapes. This reduces their ability to perform photosynthesis [71]. Furthermore, signs of hyperhydration showed low chlorophyll levels and high water content in several plants (Table 1) ([21,82]).

**Figure 4:**
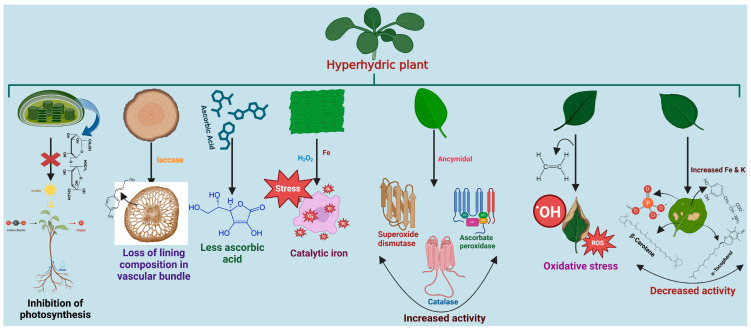
The features of the hyperhydric condition.

Lignin is an essential part of the cell wall in vascular plants, helping to build the structure of stems and leaves and protecting against environmental stresses, both biotic and abiotic. Additionally, cytological and histochemical analyses of hyperhydric shoots indicated a notable loss in lignin content and composition (Figure 4) [83]. Recent studies of two genes encoding laccases revealed that *lac4* and *lac17* are crucial in lignin polymerization. The loss of two genes in Arabidopsis caused a severe reduction in lignin content. Disrupting *lac11*, *lac4*, and *lac17* simultaneously leads to hampering plant growth and impaired vascular development [84]. RNA-Seq analysis revealed a reduced expression of laccase 3 (*ppa003408m*) in hyperhydric plant leaf tissue compared to control leaf tissue. RTqPCR data showed a 4.2-fold down-regulation of lac3 expression compared to the control. Increased phenol production in raspberries, preventing hyperhydricity, resembles plant secondary metabolite stimulation. This could relate to regulating phenolic biosynthesis, essential for lignin synthesis [9].

### 3.2. Biochemical Changes

Ascorbic acid levels in normal and hyperhydric shoots were observed in *Satix babylonica* and *Prunus avium*. Hyperhydric plantlets showed high water content and significantly less ascorbic acid (approximately one-third) than normal shoots (Figure 4). In the other two species (*Phoenix dactylifera* and *Aloe polyphylla*), the concentration of ascorbic acid stayed relatively constant [85]. In addition, large starch deposits are found in the chloroplasts of high moisture plant tissues, inhibiting their ability to photosynthesize under hyperhydric conditions (Figure 4). When photosynthesis is limited during early transplantation, these starch reserves serve as a source of carbon [21,82].

The elevated lipid peroxidation level is a result of ROS accumulation, which is generated through the reaction between H_2_O_2_ and Fe^2+^ [86]. Transition metals catalyze the formation of OH• through Fenton-type reactions. In many hyperhydric plant tissues, this iron, often called catalytic iron, accumulates [87] in their plant body systems (Figure 4). High ROS levels can induce lipid peroxidation and intensive membrane damage in hyperhydric plants [20]. In addition, the study observed that the hyperhydric blueberry plantlets showed considerable increases in the levels of ethylene and ROS, particularly H_2_O_2,_ in the guard cells. A buildup of reactive oxygen species (ROS) and water in hyperhydric plantlets raises the possibility that ethylene production, ROS creation, and water accumulation are related processes [3]. When AgNO_3_ was added to the culture media, ethylene and ROS levels dropped, guard cells accumulated H_2_O_2_, the stomatal response was changed, and water accumulated. While obtaining more H_2_O_2_ and water in guard cells, hyperhydric plantlets showed decreased stomatal aperture, density, and water loss. An oxidative burst is brought on by the accumulation of ROS, which can originate from different organelles [88].

Numerous plant species, including *Prunus avium* [89] and carnations [90], have shown a correlation between hyperhydricity and antioxidant enzymatic activities. According to studies, when hyperhydricity occurs, catalase activity is much higher in shoots cultured with ancymidol (ANC). ANC is a gibberellin-biosynthesis inhibitor whose impact depends strongly on its concentration, exposure duration, and the specific culture conditions [5]. Applying paclobutrazol in the medium with ANC reduces shoot malformation and hyperhydricity [68]. Preliminary data reveal that in the basal medium with ANC, leaf sections display considerably increased gibberellin levels, presumably explaining the shoot elongation. Increased antioxidant enzymatic activities, protein levels, and starch levels were observed in hyperhydric shoots and meristematic cluster formations in liquid-cultured plants. Franck et al. [80] showed that ascorbate peroxidase (APX), superoxide dismutase (SOD), and glutathione reductase activities were increased (Figure 4) in hyperhydric *Prunus avium* shoots. They also discovered that superoxide dismutase and catalase activity were greatest in hyperhydric shoots [85]. In addition, APX activity decreased in the hyperhydric shoots, and the medium showed signs of hyperhydricity and shoot deformity (Figure 4) [8]. The three cultivars of hyperhydric leaves had much lower PAL activity and lignin concentration than their regular leaves [91].

The increased Fe and K concentrations were found in hyperhydric shoots, and, conversely, phosphorus was remarkably found at reduced levels (Figure 4) [91]. Carotenoid concentrations varied significantly among melanocarpa leaves (Figure 4). The leaves from greenhouse-grown plants had the highest carotenoids (347.7 g), compared with normal leaves (255.2 g) and hyperhydrated leaves (109.7 g) produced from in vitro-regenerated shoots. The leaves from greenhouse-grown plants had the most tocopherol (533.3 g), followed by those from normal leaves (73.2 g) and hyperhydrated leaves (39.3 g) obtained from in vitro-regenerated shoots. These tocopherol concentrations were significantly higher than those found in berries (17.1 mg) [92].

**Figure 5:**
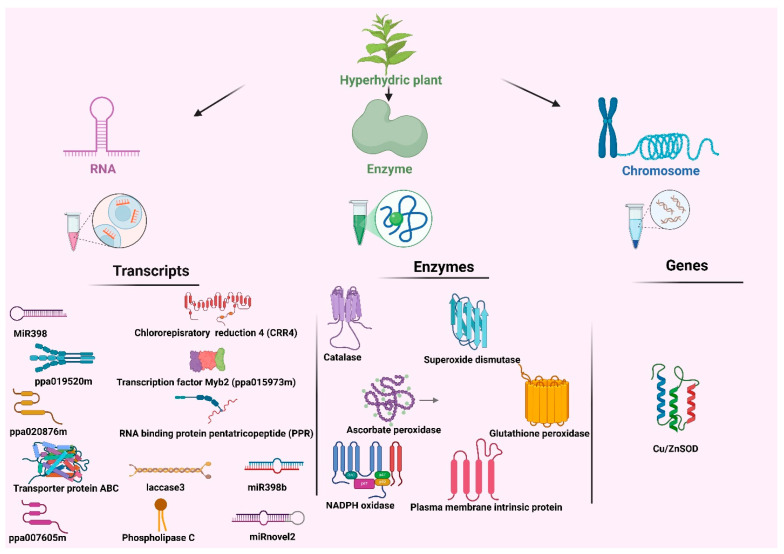
Identification of molecules in a hyperhydric plant. RNA, enzyme and chromosome indicate that each group of molecules were detected through transcript, enzyme and gene level analyses, respectively. Transcriptomic analysis of hyperhydric shoots revealed altered abundance of miR398, miR398b, miRnovel2, ppa019520m, ppa020876m, ppa007605m, chlororespiratory reduction 4 (CRR4), transcription factor Myb2 (ppa015973m), an RNA-binding pentatricopeptide repeat (PPR) protein, an ABC transporter, laccase3 and phospholipase C. Enzymatic assays showed changes in catalase, superoxide dismutase, ascorbate peroxidase, glutathione peroxidase, NADPH oxidase and plasma-membrane intrinsic proteins. Gene analysis at the chromosomal level identified Cu/ZnSOD gene found in hyperhydric plant.

The pentatricopeptide (PPR) protein, which is responsible for the post-transcriptional control of organelle gene expression and photosynthesis, is encoded by two down-regulated transcripts (ppa019520m and ppa020876m) among the top 30 DETs (differentially expressed transcripts) (Figure 5) observed in hyperhydric plantlets.

## 4. RNA Sequencing and HPLC-Based Metabolite Profiling for Hyperhydric Plants

RNA-Seq and HPLC analyses revealed the active genes and metabolite characteristics in hyperhydric plants. RNA sequencing (RNA-Seq) analysis revealed differences in the expression of more than 300 transcripts between control and hyperhydrated leaf cells. The expression of numerous transcripts, including transcription factor Myb2, RNA-binding protein pentatricopeptide (PPR), transporter protein ABC, and laccase 3, was altered (Figure 5) in hyperhydric plantlets. The expression of miRNAs is altered by hyperhydricity in the peach, and miR398b down-regulation may be related to an abiotic stress response (Figure 5) [9]. The top 20 transcripts showing the most significant differential expression, indicating hyperhydricity, were classified as having an unknown function. However, out of the ten transcripts known to induce hyperhydricity, four of them, namely alcohol dehydrogenase (ppa007605m), MYB2 (ppa015973m), laccase 3, and phospholipase C, were found (Figure 5) to be statistically significant at the *p* < 0.05 level. Among these, the expression of two transcripts (ppa007605m and ppa015973m) was higher in peach’s hyperhydric leaf tissue than in the control [9]. A novel miRNA called miRnovel2 inhibits in hyperhydric leaves and may target the transcript of gibberellin 2-beta-dioxygenase. Most transcripts regulated by miRNAs exhibit catalytic activities, including oxidoreductase function. MiR398 down-regulation is one source of them (Figure 5) [93]. During in vitro culture, hyperhydricity was tested in transgenic potato plants that either carried the sense or antisense lily chloroplastic *Cu/Zn-SOD* gene [94]. In addition to high H_2_O_2_, there was a high level of ethylene buildup in culture vessels, where the *Cu/Zn-SOD* gene was probably responsible for the development of hyperhydricity. Our analysis shows that antisense expression of the *Cu/Zn-SOD* gene reduces hyperhydricity in micropropagated potato plants (Figure 5) [94].

High-performance liquid chromatography (HPLC) has emerged as a pivotal tool in profiling bioactive compounds associated with hyperhydricity in in vitro-cultured plant tissues, although its use remains underrepresented in current research. Hyperhydricity leads to altered metabolism, oxidative stress, and aberrant hormone signaling, often linked to disrupted secondary metabolite biosynthesis, which HPLC can sensitively detect. As shown in Table 2, HPLC enabled the identification of hyperhydric stress-related compounds such as ecdysteroids [95], vincristine [96], vinblastine [96], rosmarinic acid [97], and endogenous cytokinins [98] in hyperhydric tissues of Catharanthus, Thymus, Aloe, and others. These findings underscore the diagnostic significance of HPLC in uncovering metabolism under hyperhydric conditions, such as cytokinin regulation and many more. For instance, the accumulation of rosmarinic acid was notably enhanced under low TDZ levels and silver nitrate treatment, suggesting both a stress response and a potential recovery compound [96,97]. Furthermore, endogenous cytokinin profiling in *Aloe polyphylla* (Table 2) confirmed that increased cytokinin levels were induced by gelrite, reinforcing the relevance of hormonal imbalance under hyperhydric conditions [11]. Despite these insights, HPLC applications remain limited across species, pointing to a critical gap in understanding the biochemical hallmarks of hyperhydricity. Systematic metabolite profiling using targeted HPLC panels could enhance early detection, standardize phenotyping, and support the development of hyperhydricity control in commercial micropropagation systems [3,99].

## 5. Ultrastructural Changes

Normal blueberry and hyperhydric blueberry plants showed significant variations in cellular and tissue structure; the scanning electron microscope and transmission electron microscopy were able to identify these differences. The epidermal abaxial surface of leaves was observed using the SEM. The epidermal surface was found to have regular stomata, with the remaining epidermal cells in normal in vitro plants [6]. However, in hyperhydric plants, the guard cells were observed to be larger due to the excessive water absorption tendency, eventually leading to turgidity and a transformed cell wall structure. Furthermore, deformation and torn places were investigated in some areas of hyperhydric leaves. The analogous result was reported by Fontes et al. [71] for hyperhydric *Datura insignis* plants. On the other hand, the deformation was underlined by the loss of elasticity or cellulose microfibril deposition. Plastoglobulin and some starch granules were seen in the stromal region between the thylakoid membranes. These structures build up because of chloroplast aging, poor nutrition, herbicide application to crops, and other stresses. *Eucalyptus grandis* and *E. urophylla* were followed by Louro et al. to observe an increase in plastoglobulin density in plants cultivated on a stretch root medium [103].

## 6. Artificial Intelligence Model for Optimizing the Algorithm in Hyperhydricity

Digital solutions for complex labor processes are now a part of the agricultural industry due to advancements in sensor technology, automation, and robotization and data analysis using traditional and cutting-edge machine learning (ML) techniques. AI models serve three key functions in tissue culture and specifically in managing hyperhydricity. First, they enable diagnostic insight: by training models on input variables (endogenous hormonal balance and effect of nutrients) and output variables (exogenous hormonal balance, temperature, gelling agents) to keep endogenous hormonal balance between different cell types, researchers can identify the effect of hyperhydricity and focus the strategies more effectively. Here, the endogenous hormonal balance regulates differential growth among cell types. Since exogenous PGRs act as cofactors that modify endogenous hormone activity, their concentrations, along with nutrient composition, are included as core inputs. Second, AI provides optimization support for optimizing the combinations of culture variables such as gelling agent concentration, vessel sealing type, and growth hormone dose to minimize hyperhydricity occurrence while maintaining shoot proliferation and quality [104]. Third, AI facilitates scalable and reproducible workflows when developing the culture condition; predictive models reduce the need for empirical testing, support real-time monitoring (via imaging or sensors), and can integrate into automated or semi-automated systems for large-scale micropropagation. Moreover, AI with digital imaging and sensor-based monitoring enables the real-time observation and adjustment of in vitro environments. Ultimately, AI tools in tissue culture research enhance reproducibility and cost efficiency as well as supporting scalable commercial production of high-quality, physiologically normal plants [105].

**Figure 6:**
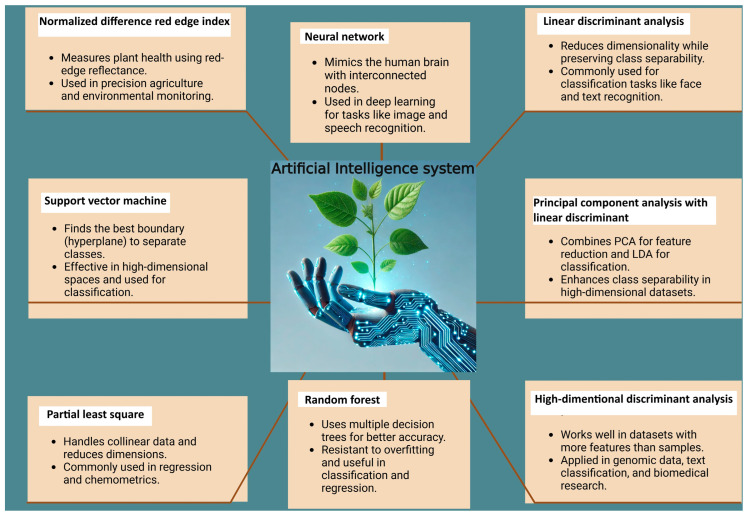
Different artificial intelligence systems for the accuracy of hyperhydricity identification.

Many machine learning models exist, including NNET (neural network), LDA (linear discriminant analysis), SVM (support vector machine), RF (random forest), PLS (partial least squares), and HD.DA (high dimensional discriminant analysis), PCA.LD (principal component analysis with linear discriminant), and NDRI (normalized difference red edge index) (Figure 6). Those AI models analyze complex interactions among in vitro culture parameters such as light, humidity, cytokinin levels, vessel aeration, and media composition for the measurement of hyperhydricity. These methods have been shown to accurately predict outcomes such as rooting percentage, shoot proliferation, and incidence of physiological disorders (e.g., vitrification, necrosis) [106,107,108]. Moreover, AI algorithms apply systematically across tissue culture stages, enabling researchers to move from empirical trial and error to predictive modeling and protocol optimization (e.g., media design, environmental control) [109]. In hyperhydricity, which involves morphological, anatomical, and metabolic alternation in vitro [85], AI models can facilitate early detection (via imaging or spectral data), prediction of risk based on culture parameters, and adjustment of conditions to minimize incidence. According to Bethge et al. [105], the MLMs varied in architecture, complexity, performance, prediction time, and interpretability. With a test accuracy of 85%, SVM performed better than other models, and the traditional vegetation index classification method was evaluated by a machine learning model (Figure 6) [105]. SVM was chosen due to its superior performance on training and test datasets, low volume training data requirements, performance on high-dimensional datasets, low risk of overfitting, good generalization ability, and advantages over NNET in terms of training time and interpretability. Based on their investigation, Bethge et al. established hyperhydricity detection using an RGB camera system in conjunction with a deep neural network (DNN) and offered two potential solutions for industrial plant propagation [105].

## 7. Preventive Measures for Hyperhydricity

**Figure 7:**
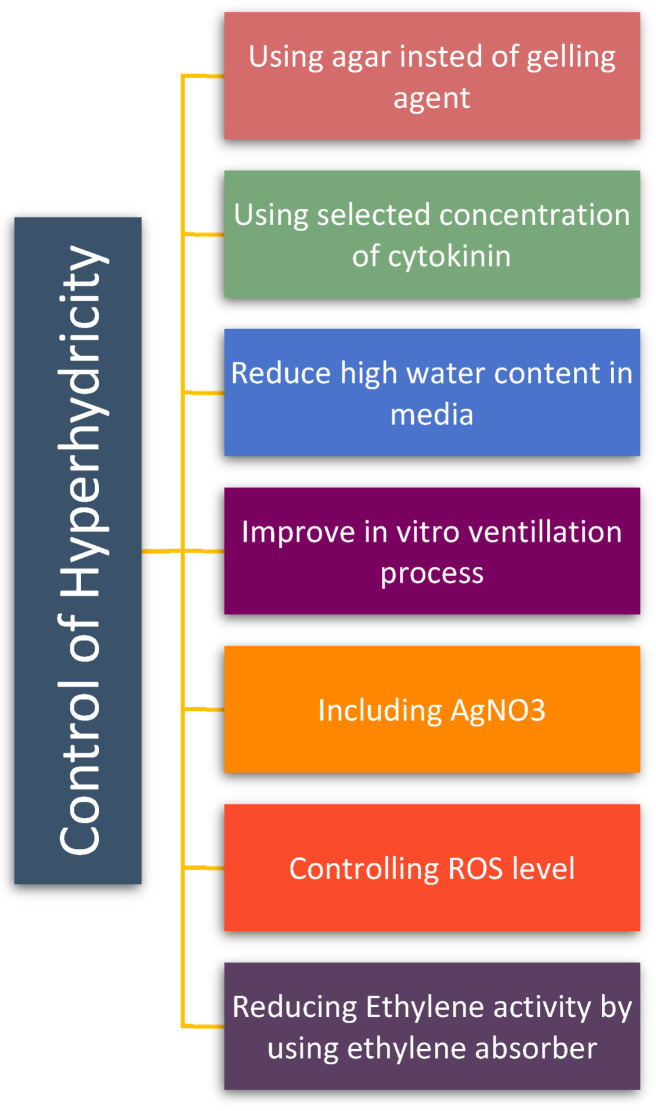
Control of hyperhydricity.

Hyperhydricity damages reproduction, and it causes rising water, which is a significant concern (Figure 7). The addition of thidiazuron (TDZ) decreases the frequency of hyperhydricity. As a result, reducing the level of cytokinins in the medium could be an additional method for lowering hyperhydricity. Several recovery strategies were followed using ethylene absorbers, ethylene biosynthesis inhibitors, and free radical scavengers on explants. Among them, reduced ethylene activity (Figure 7) in in vitro blueberries decreased the incidence of hyperhydricity. For instance, applying ethylene absorbers such as charcoal [110], KMnO_4_, or alginate capsules [111] eliminated high water content. In addition, AVG (aminoethoxyvinylglycine), an inhibitor that prevents ethylene production, was added to the medium to decrease the quantity of vitrified *Ixoracoccinea* L. buds [112]. Cultivation of Dianthus chinensis L. seedlings on medium supplemented with AgNO_3_ facilitates the recovery of highly hydrated seedlings to a normal growth. Additionally, the use of inhibitors like silver nitrate, which block ethylene activity, has been studied in various plants such as *Dianthus chinensis* L. [36], watermelon [31], sunflower [7], and *Ziziphus jujuba* Mill [113]. AgNO_3_ addition to the medium allowed hyperhydrated explants in micropropagated plants to transferred to normal growth, minimizing economic losses due to hyperhydricity [54]. Additionally, several plant growth regulators protect against environmental stress, such as polyamines (PAs) and salicylic acid (SA) [57]. Exogenous application of polyamines (PAs) and their precursors significantly reduced hyperhydricity in micropropagated apple and carnation shoots, indicating that PAs can help to regain endogenous growth regulators and redox balance in hyperhydric plants [30,46]. In addition, salicylic acid (SA) reversed hyperhydricity in *Thymus daenensis* while shifting endogenous polyamine pools, indicating that exogenous SA can help restore hormonal homeostasis [57]. H_2_O_2_ was avoided by employing agar as the gelling agent (Figure 7). However, in blueberry tissue culture, hyperhydric shoots exhibited the highest catechin content [110]. In *Aloe polyphylla*, adjusting the type and concentration of exogenous cytokinins lowered the incidence of hyperhydricity while altering endogenous cytokinin (CK) profiles, showing that carefully optimized CK supply can normalize internal hormone balance in shoot cultures [28,73,98]. Similarly, cytokinin controlled the proliferation and hyperhydricity reported in pear and *Thymus leucotrichus*, where specific CK induced non-vitrified shoots and normalized the auxin–cytokinin equilibrium by external regulators [64,97]. In blueberry and lingonberry, optimizing liquid in a temporary immersion bioreactor improved antioxidant status and reduced hyperhydricity and canalization in *Vaccinium* spp. [3,22,99]. Developmental studies in mangosteen leaf tissues showed that auxin, cytokinin, and ethylene differentially regulate specific stages of bud morphogenesis, induce hormone canalization, and thereby alter susceptibility to hyperhydricity [1,72,112]. Medium optimization using modeling and machine learning approaches has revealed that specific combinations of minerals, carbohydrates, and PGRs are critical to minimize physiological disorders such as hyperhydricity by driving endogenous hormonal profiles into the control of hormone canalization [17,85,106,107,108,109].

## 8. Conclusions

The stressed explants release ethylene and ROS during micropropagation. The accumulation of H_2_O_2_ in guard cells destroys their structure and makes stomata move unevenly if the plant’s antioxidant system does not suppress ROS. Due to an imbalance between water intake and tissue water loss, more water builds up in plant tissues. Excessive water buildup in the tissue ultimately leads to increased hypoxic stress, seriously harming the cell structure. By lowering ethylene activity and limiting the buildup of ROS, hyperhydricity may be prevented. Hyperhydration is reflected at the molecular level by changes in the expression of several transcripts (around 300) identified by RNA-Seq. The genome-wide study will help identify the underlying molecular mechanisms of H_2_O_2_ that cause substantial financial losses in fruit production. AgNO_3_ restored hyperhydricity by reducing ethylene and H_2_O_2_ accumulation and increasing water loss. These findings demonstrate that excessive ethylene accumulation in plant tissues significantly contributes to the development of hyperhydricity and supports the role of AgNO_3_ in treating hyperhydricity condition improvement. Biochemistry and molecular biology studies demonstrated that overhydrated tissues experienced oxidative stress, ultimately leading to the death of in vitro explants at the commercial level.

## Figures and Tables

**Table 1:** Hyperhydricity observed in in vitro-cultured plants.

Species	Plant Material	Type of In Vitro Culture	Major Observations	Reference
Agave (*Agave sisalana* Perrine)	Central meristematic part of the bulbils	MS medium	Include osmotic stress-causing substances like sodium chloride and polyethylene glycol (PEG 6000) to solve the hyperhydricity problem	[42]
Apple (*Malus domestica* Borkh. cv M9 EMLA)	Rootstocks	MS medium	Treatment with 60 mM maltose and 30 mM sorbitol triggered hyperhydricity, accompanied by a decrease in chloroplast abundance, enhanced activity of antioxidant enzymes, and a higher proportion of oxidized pyridine nucleotides	[43]
Arabidopsis (*Arabidopsis thaliana* (L.) Heynh (Col-0)	Seeds	½ MS salt medium	Water accumulated heavily in the apoplast of the leaves of hyperhydrated seedlings and flooded the apoplastic air spaces; the increased expression of hypoxia-responsive genes in hyperhydrated seedlings revealed that the water saturation of the apoplast decreased oxygen supply; impairment of gas exchange reduced stomatal aperture in hyperhydrated plants	[1]
Blueberry (*Vaccinium* spp.)	Young stems	½ MS salt with 2.0 mg zeatin	Hyperhydric plantlets exhibited reduced cell wall thickness, decreased number of mitochondria, excessive accumulation of ROS and ethylene, abnormality of stomatal movement	[3]
Cactus (*Mammillaria gracilis* Pfeiff.)	Roots	MS medium	The spines of the hyperhydric shoots showed elongated trichomes	[44]
Caladium (*Caladium bicolor* (Aiton) Vent. cv. “Bleeding hearts”)	Rhizomes	MS medium supplemented with 2, 4-dichlorophenoxy acetic acid	Hyperhydricity was more frequent in liquid over solid medium-cultured plantlets and often happened in shoot cultures, influenced by the photoperiodic incubation and culture conditions	[14]
Cancer bush (*Lessertia frutescens*) (L.) Goldblatt & J.C. Manning)	Nodal segments	MS medium	In the temporary immersion bioreactor, 50% displayed hyperhydricity signs	[45]
Carnation (*Dianthus caryophyllus* L.)	Shoots	MS medium	Red and blue light reduced hyperhydricity	[23]
Carnation plant (*Dianthus caryophyllus* L. var. Nora)	Shoots	MS medium (liquid or semi-solid)	Stomatal malfunction in response to light and dark. Hyperhydric plantlets showed oxidative damage, high malondialdehyde (MDA) content, low lignification, and high peroxidase activity	[46]
Carnation cv. White sim, Exquisite and Scania (*Dianthus caryophyllus* L.)	Nodal segments	MS medium	Iron and/or magnesium in the media reduced hyperhydricity	[47]
Carnation (*Dianthus caryophyllus* cv. Killer)	Shoots	MS medium	Disrupted middle lamella and the intercellular spaces	[48]
Carnation (*Dianthus caryophyllus* L. cv. Nelken)	Nodal segments	MS medium	Hyperhydric tissues exhibit poorly developed cell walls, a less dense cytoplasm, underdeveloped chloroplasts, an increased vacuolar area, and structural abnormalities in stomata	[49]
Carnation (*Dianthus caryophyllus* L. cv. Oslo, Killer and Alister)	Shoots	MS medium	Hyperhydric leaves have reduced pectins and higher (4–10 times) pectin methyl esterases activity	[50]
Chinese peony (*Paeonia lactiflora* Pall. ‘Zhong Sheng Fen’)	Buds	½ MS medium containing double calcium chloride	Hyperhydric microshoots were successfully revived and allowed to develop normally by adding activated charcoal, removing ammonium nitrate from the media, doubling the content of Ca^2+^, or removing BA from the medium	[51]
Coffee (*Coffea arabica* L.)	Young leaves	½ MS callogenesis medium	Higher water content, more negative water potential values, and higher K^+^ concentration were all characteristics of hyperhydric embryos; the most torpedo-shaped embryos lacking hyperhydricity were produced using 1 min immersions every 4 h, and they were successful in regenerating plants (75% of the time)	[52]
Common centaury (*Centaurium erythraea* Rafn)	Shoot tips	MS medium (liquid) with 6-benzylaminopurine and indole-3-acetic acid	More shoot production but less vigor	[53]
China pink (*Dianthus chinensis* L.)	Nodal segments	MS medium	Lowered the levels of hydrogen peroxide (H_2_O_2_)	[54]
Cotoneaster (*Cotoneaster wilsonii* Nakai)	Nodal and shoot-tip explants	MS medium containing 2-isopentyl adenine, 6-benzyladenine, and thidiazuron alone or in a combination with naphthaleneacetic acid	Hyperhydric shoots were produced by high TDZ concentrations and repeated subcultures	[55]
Crown-of-thorns (*Euphorbia milii* Des Moul.)	Apical meristems	MS medium (liquid) in temporary immersion bioreactor	Increase in malondialdehyde, dehydroascorbate reductase, glutathione reductase, catalase, ascorbate peroxidase, peroxidase, monodehydroascorbate reductase, and superoxide dismutase enzymes	[8]
Date palm (*Phoenix dactylifera* L. cv Al-Fayda)	Adventitious buds	½ MS medium (liquid)	Different concentrations of the exogenous hormones: 2iP 6-(dimethylallylamino) purine, 6-benzylaminopurine, indole-3-acetic acid, indole-3-butyric acid, kinetin, 2-naphthoxyacetic acid resulted in varying frequencies of hyperhydricity and finally led to tissue browning	[56]
Denaian thyme (*Thymus daenensis* Celak.)	Seeds	MS medium	Chlorophyll deficiency in shoots and reduced differentiation during growth	[57]
Eggplant (*Solanum melongena* L.)	Germinated seedlings	MS medium	Inducing a higher accumulation of binding protein	[58]
Eucalyptus hybrids and species (*Eucalyptus grandis* W.Hill; *E. camaldulensis* Dehnh.; *E. urophylla* S.T.Blake; *E. dunnii* Maiden)	Axillary shoots	MS medium (liquid)	Enhanced absorption of cytokinin led to the suppression of apical dominance	[24]
Figwort (*Scrophularia yoshimurae* T.Yamaz)	Nodal segments	MS medium	Proper ventilation in liquid culture reduced hyperhydricity than sealed parafilm	[59]
Garlic (*Allium sativum* L. cv. *Ershuizao*, *Cangshan* and *Zhengyuezao*)	Shoots	MS medium	Younger inflorescence and smaller explant	[10]
Garlic (*Allium sativum* L. cv. *Ershuizao*)	Bulbs	B5-based MS medium	Chloroplast and mitochondrial ultrastructure are disrupted	[18]
Gladiolus (*Gladiolus hybridus* Hort. cv. Wedding Bouquet)	Primary leaves of the sprouted corms	MS medium	Maximum shoot multiplication with minimal to no hyperhydricity symptoms was achieved using liquid culture	[60]
Grape (*Vitis vinifera* L. cv. Cabernet sauvignon)	Axillary shoot tips	½ MS medium with 6-benzyladenine	Dehydration improved shoot recovery for hyperhydricity	[61]
Grapple (*Harpagophytum procumbens DC. ex Meisn.*)	Shoots	MS medium with sucrose and 6-benzylaminopurine	Silicon reduced shoot-tip necrosis, improved leaf and shoot growth, and increased root stability at their base	[62]
Horse chestnut (*Aesculus hippocastanum* L.)	Stems	WPM	Cytokinin (6-benzyladenine and thidiazuron) increased hyperhydricity	[63]
Japanese pear (*Pyrus pyrifolia* (Burm.f.) Nak. cv. Hosui and Kosui)	Shoots	WPM	Thidiazuron stimulated hyperhydricity	[64]
Japanese pear (*Pyrus pyrifolia* (Burm.f.) Nak. cv. Hosui)	Shoots	½ MS with indole-3-butyric acid and 6-Benzyladenine	Charcoal in the medium increased shoot hyperhydricity	[65]
Jojoba (*Simmondsia chinensis* (Link) Schneider)	Seedlings	Multiplication medium supplemented with benzylaminopurine	Numerous morphological flaws were evident in hyperhydric plantlets, including hypertrophy of the stem cortex and mesophyll, improperly shaped non-functional stomata, epidermal discontinuity, and xylem hypolignification; they did not survive during acclimatization	[66]
Lingonberry shoots (*Vaccinium vitis-idaea* L.)	Shoots	Debnath and McRae’s basal medium	Zeatin (9.1 μM) induced higher hyperhydricity	[22]
Mango (*Mangifera indica* L.)	Ovule	MS medium	Abscisic acid reduced the hyperhydricity of the primary somatic embryo	[67]
Narcissus (*Narcissus tazetta* L. cv. *Ziva*)	Bulbs	MS medium (liquid)	Ascorbate peroxidase and catalase activities were decreased	[68]
Olive (*Olea europaea* L.)	Nodal segments	WPM	Replacing the gelling agent with agar prevented the occurrence of hyperhydricity	[69]
Oregano (*Origanum vulgare* L.)	Germinated seedlings	MS medium	The application of *Pseudomonas* spp. in in vitro *Origanum vulgare* culture significantly reduced hyperhydricity	[70]
Peach (*Prunus persica var. nectarina* (Aiton) Maxim.)	Shoots	MS medium	Hyperhydricity modifies the expression of numerous transcripts, including the transporter protein ABC, the transcription factor Myb2, the RNA-binding protein pentatricopeptide, and the laccase 3 mRNA	[9]
Pepper (*Capsicum annuum* L. cv ‘Agronômico G10′)	Seeds	MS medium lacking growth regulators	The chloroplasts showed thylakoid disorder, low grana number, an accumulation of large starch grains, and a low accumulation or lack of plastoglobulus, which is an 80 kDa protein generated in hyperhydric plants	[71]
Pink lapacho (*Handroanthus impetiginosus* (Mart. ex DC) Mattos)	Unrooted shoots	MS salt medium	Collapsed cells, epidermal holes, epidermal discontinuity, disorganized cortex	[40]
Potato shoot (*Solanum tuberosum* L.)	Tubers	Completely sealed vessel	No ethylene found; lower dry weight and chlorophyll content	[72]
Red beet (*Beta vulgaris* var. Conditiva)	Germinated seeds	MS medium	The application of Deuterium-depleted water (DDW—25 ppm deuterium) in in vitro culture prevents hyperhydricity	[33]
Spiral aloe (*Aloe polyphylla* Schonland ex Pillans)	Germinated seeds	MS medium	Gerlite induces higher hyperhydricity	[11]
Spiral aloe (*Aloe polyphylla*)	Germinated seeds	MS medium	Natural ventilation effectively reduces hyperhydricity	[16]
Spiral aloe (*Aloe polyphylla*)	Germinated seedlings	MS medium	Ammonium cytokinins and ions induce hyperhydricity	[28]
Spiral aloe (*Aloe polyphylla*)	Germinated seedlings	MS medium	NO_3_^−^ in the media reduced hyperhydricity	[73]
Sugar beet (*Beta vulgaris* L. cv. Felicita)	De-coated seeds	MS medium supplemented with polyethylene glycol 6000	Hyperhydricity levels increased along with browning and/or blackening of tissues in culture when polyethylene glycol 6000 was exposed for prolonged periods (up to 4 weeks); several antioxidant enzyme activities increased, notably at lower polyethylene glycol 6000 concentrations	[74]
Sunflower (*Helianthus annuus* L.)	Shoots	MS medium	Silver nitrate including media reduced hyperhydricity	[7]
Thapsia (*Thapsia garganica* L.)	Petiole and leaflet	MS medium	2% polyethylene glycol with adequate ventilation decreases hyperhydricity	[75]
Thimble cactus (*Mammillaria gracillis* Pfeiff.)	Leaves	MS medium	Without using hormones, shoots naturally formed calluses that produced normal and hyperhydrated nodes	[76]
Tiepishihu *(Dendrobium officinale* Kimura & Migo)	Nodal segments	MS medium	Polyethylene glycol 6000 significantly hampers water metabolism and the antioxidant system	[77]
Triploid watermelon [*Citrullus lanatus* (Thunb.) Matsum. and Nakai]	Seeds	Basal MS medium	High 6-benzyladenine resulted in multiple shoots and increased hyperhydricity; proper ventilation with cotton bunks or cellulose nitrate filters reduced hyperhydricity	[78]
Watermelon (*Citrullus lanatus* Thunb.)	De-coated seeds	MS medium	Silver nitrate supplementation led to the highest decrease in hyperhydricity and an increase in the number of shoots	[31]
Watermelon (*Citrulus lanatus* cv. Giza 1)	Seeds	MS medium containing 6-benzyladenine, kinetin or thidiazuron	6-benzyladenine concentration (above 2 mg/L) resulted in many shoots and more hyperhydricity, but vessel aeration and medium substitution of gelrite for agar decreased hyperhydricity symptoms	[79]
Wild cherry (*Prunus avium* L.)	Shoots	MS medium	Elevated levels of stress indicators, including ethylene, polyamines, and proline	[80]
Yanhuanglian (*Corydalis saxicola* Bunting)	Leaves	MS medium containing 6-benzyladenine and naphthaleneacetic acid	Cytokinins such as 6-benzyladenine and thidiazuron causing hyperhydricity during shoot organogenesis	[81]

Abbreviations: Murashige and Skoog (MS); woody plant medium (WPM).

**Table 2:** Compound observations at hyperhydric plants.

Plant Part	Method	Compound	Findings	Reference
Flowering herb and roots of Ragged Robin	HPLC	Ecdysteroids	Adventitious roots are stirred up; culture can be thought of as a different source of biomass that is high in pharmacologically active ecdysteroids	[95]
Catharanthus leaf sample	HPLC	Vincristin, vinblastine, and total alkaloid	Medicinally important compounds were found (vincristine, vinblastine, and total alkaloid)	[96]
Common buckwheat seeds	NMR and HPLC	Rutin, quercetin	When exposed to blue light, rutin and quercetin extracts were obtained at 4.3 mg and 7.0 mg/g, respectively, as opposed to 3.7 mg of rutin/g of extract and traces of quercetin	[100]
Seeds of Moonlight Thyme	HPLC	Rosmarinic acid	The lowest concentration of thidiazuron (0.1 mg/L) was adequate for high synthesis of rosmarinic acid (9.25 mg/g). AgNO_3_ applied to reduce hyperhydricity	[97]
Potato plants	HPLC	Zeatin-type cytokinins	Media supplemented with jasmonic acid to reduce the production of cytokinins	[101]
Shoot culture of Aloe	HPLC	Endogenous cytokinins	Up-regulation of endogenous cytokinin levels is at least largely responsible for exogenous cytokinins’ and gelrite’s ability to induce hyperhydricity in shoots of *Aloe polyphylla*	[98]
Aerial parts of *Echinacea angustifolia D.C.*	LC–DAD–ESI–MS	Alkamides, flavonoids, and caffeolquinic derivatives	To improve the production, growers and herbal product companies may find it quite convenient to clone selected superior individuals using flower stalk explants	[102]

## Data Availability

No new data were created or analyzed in this study. Data sharing is not applicable to this article.
